# Anti-Biofilm Activity of (+)-Endo-Borneol Against *Streptococcus mutans*: Experimental Evaluation, Virulence Gene Expression Analysis, and Molecular Docking

**DOI:** 10.3390/plants15162417

**Published:** 2026-08-07

**Authors:** Gayane Atazhanova, Karakoz Badekova, Yana Levaya, Assel Sabiyeva, Tomas Kacergius, Vika Gabe, Irina Kadyrova, Altyn Bakenova, Almagul Makhmutova, Daniyar Sadyrbekov, Assanali Ainabayev, Elina Smagulova

**Affiliations:** 1 School of Pharmacy, Karaganda Medical University, Gogol Street 40, Karaganda 100017, Kazakhstan; atazhanova@qmu.kz (G.A.); ylevaya@qmu.kz (Y.L.); sabievaa@qmu.kz (A.S.); ikadyrova@qmu.kz (I.K.); bakenovaa02@gmail.com (A.B.); mahmutova@qmu.kz (A.M.); elinasmagulova0509@gmail.com (E.S.); 2 Department of Physiology, Biochemistry, Microbiology and Laboratory Medicine, Institute of Biomedical Sciences, Faculty of Medicine, Vilnius University, M. K. Ciurlionio Street 21, 03101 Vilnius, Lithuania; tomas.kacergius@mf.vu.lt (T.K.); vika.gabe@mf.vu.lt (V.G.); 3 Engineering Laboratory “Physicochemical Research Methods”, Buketov Karaganda National Research University, Mukanova Street 41, Karaganda 100024, Kazakhstan; daniyar81@gmail.com (D.S.); aliopel_82t@mail.ru (A.A.)

**Keywords:** antibiofilm activity, *Streptococcus mutans*, (+)-endo-borneol, *Achillea millefolium*, dental caries, gene expression analysis, monoterpenes

## Abstract

*Streptococcus mutans* is the primary etiological agent of dental caries due to its ability to form acidogenic biofilms on tooth surfaces. Natural monoterpenes have attracted considerable interest as potential antibiofilm agents for oral healthcare. The present study investigated the antibiofilm activity and possible mechanism of action of (+)-endo-borneol isolated from the essential oil of *Achillea millefolium* against *S. mutans*. The chemical composition of the essential oil was characterized by gas chromatography–mass spectrometry (GC–MS), and (+)-endo-borneol was isolated by chromatographic separation. Antibiofilm activity was evaluated using the crystal violet biofilm assay, while antimicrobial activity was determined by minimum inhibitory concentration (MIC) and minimum bactericidal concentration (MBC) assays. The influence of subinhibitory concentrations of (+)-endo-borneol on the expression of the biofilm-associated genes *gtfB* and *yycF* was assessed by quantitative real-time PCR. Molecular docking was performed to investigate ligand–protein interactions, using a ligand geometry pre-optimized by density functional theory (DFT, B3LYP/6-31G**). The essential oil inhibited *S. mutans* biofilm formation by up to 98%, whereas isolated (+)-endo-borneol reduced biofilm biomass by 97–98% at concentrations of 2–10 mg/mL. The MIC and MBC values of (+)-endo-borneol were 2.5 and 5.0 mg/mL, respectively. Gene expression analysis demonstrated that subinhibitory concentrations of (+)-endo-borneol modulated the transcription of *gtfB* and *yycF*, indicating activation of bacterial regulatory responses. Molecular docking revealed favorable binding of (+)-endo-borneol to biofilm-related protein targets. These findings demonstrate that (+)-endo-borneol is a promising natural antibiofilm compound with potential application in the development of novel preventive and therapeutic oral healthcare products targeting *S. mutans* biofilms.

## 1. Introduction

Among the most important classes of natural compounds, the study of which has a significant impact on the development of modern organic and bioorganic chemistry as well as the chemistry of physiologically active substances, isoprenoids occupy a prominent place. Biologically active mono- and sesquiterpenoids of plant essential oils (EO) are considered a promising group, presenting practical interest as a potential source of new pharmaceuticals [1,2,3,4,5,6,7,8].

Research on the chemistry of plant EOs, specifically their compositional analysis, conducted in the second half of the 19th century and the first half of the 20th century, contributed to the development of methods for the isolation and identification of monoterpenoids and sesquiterpenoids, the study of the fine structure of their molecules, as well as organic chemistry in general. However, the existing raw material base of accessible monoterpenoids and sesquiterpenoids from the EOs of Kazakhstan’s plants is practically unused as a renewable chemical resource for the creation of new pharmaceuticals.

The flora of Kazakhstan comprises more than 6000 species of higher plants, 677 of which are endemic—that is, growing exclusively in this region—and most of them have hardly been studied for their content of biologically active substances. We have studied the component composition of EOs from 125 plant species of the flora of Kazakhstan using gas chromatography-mass spectrometry [9,10,11,12,13], and from some of them, major components such as 1,8-cineole **1**, camphor **2**, α- and β-pinene **3–4**, linalool **5**, thymol **6**, carvacrol **7**, pulegone **8**, chamazulene **9**, etc. [14,15,16].

Numerous studies have shown that EOs and their individual components exhibit a broad spectrum of biological activity, including antibacterial, antifungal, antiviral, antitumor, anti-inflammatory, and other activities [17,18,19]. In recent years, particular attention has been paid to studying the antimicrobial activity of natural terpenoid compounds against oral microorganisms involved in the formation of dental plaque and the development of dental diseases.

The bacterium *Streptococcus mutans* plays a key role in the development of caries; it is capable of adhering to the surface of tooth enamel, forming stable biofilms, and producing organic acids that cause demineralization of hard dental tissues. In this regard, the search for natural compounds capable of inhibiting the growth of cariogenic microorganisms and suppressing the formation of bacterial biofilms represents a current focus of modern pharmaceutical and medicinal chemistry. We have identified a number of EOs from plants growing in Kazakhstan that inhibit the formation of *S. mutans* biofilms [20,21,22,23].

Of particular interest in this regard are the monoterpenoids of plant EOs, which possess pronounced antimicrobial and membrane-targeting properties. One such compound is borneol **10**—a bicyclic monoterpene alcohol widely found in the EOs of many aromatic plants. In this study, the subject of investigation is borneol isolated from the EO of *Achillea millefolium*. Given the known antimicrobial properties of this compound, it appears promising to investigate its inhibitory effect on the growth and biofilm formation of cariogenic bacteria, which may contribute to the development of new caries prevention agents.

**Figure 1:**
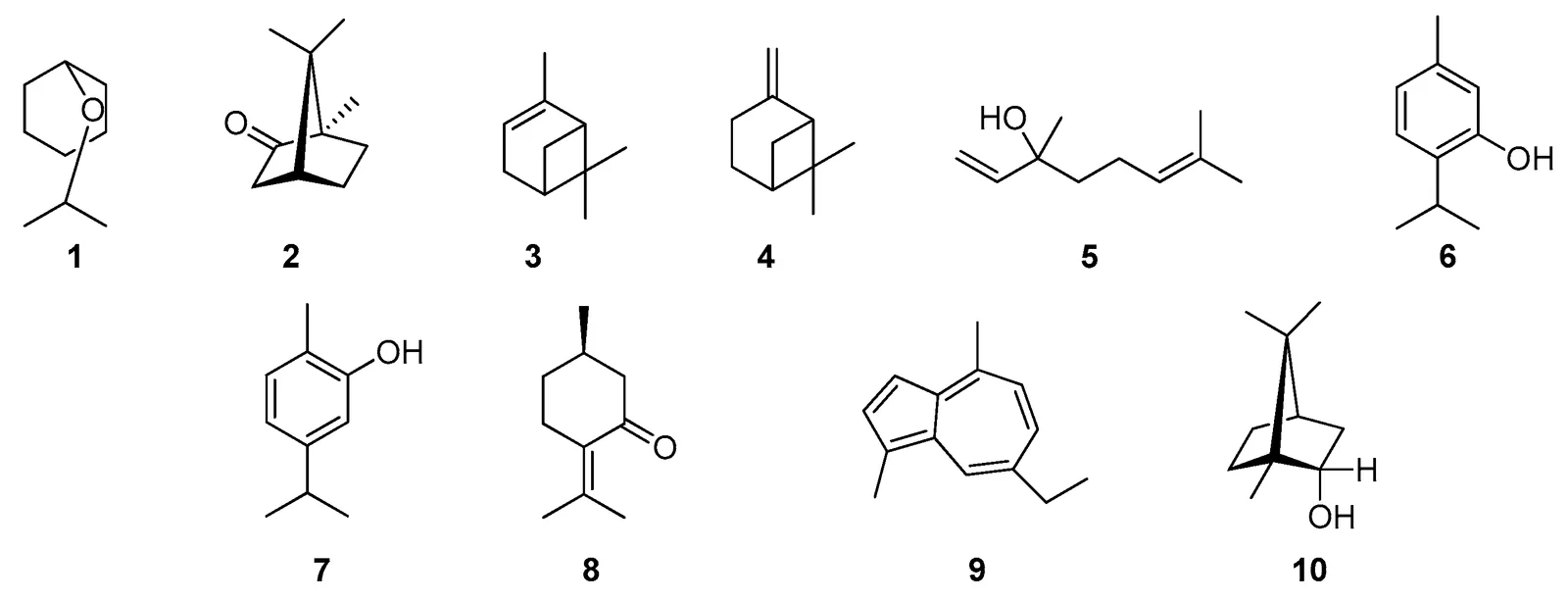
Structure of the most common mono- and sesquiterpenoids in essential oils of *Achillea* species.

The genus *Achillea* of the family *Asteraceae* comprises 11 species in Kazakhstan [24]. As part of our research, we isolated EOs from nearly all species of this genus growing in Kazakhstan and analyzed their chemical composition using gas chromatography–mass spectrometry [25,26]. It has been established that the EOs of *Achillea* species are characterized by a complex chemical composition and contain significant amounts of mono- and sesquiterpenoids, such as 1,8-cineole **1**, camphor **2**, borneol **10**, and other components (Figure 1).

Among the representatives of this genus, the plant *A. millefolium* is of particular interest; it is widely distributed in various regions of Eurasia and is known as a valuable medicinal plant. Numerous studies have shown that *A. millefolium* possesses a broad spectrum of pharmacological activity, including anti-inflammatory, antibacterial, antioxidant, antispasmodic, and wound-healing effects [27,28,29].

The pharmacological properties of this plant are largely due to the presence of an EO rich in monoterpene compounds. During the fractionation of *A. millefolium* EO to isolate major components such as 1,8-cineole 1 and camphor 2, we also isolated a concomitant monoterpene alcohol—borneol **10**—which is of interest due to its biological activity [30,31,32].

This study aimed to isolate (+)-endo-borneol from the essential oil of *A. millefolium* and to characterize its antibiofilm activity against *S. mutans*. The impact of (+)-endo-borneol on the expression of biofilm-associated virulence genes was further evaluated. To gain mechanistic insight, molecular docking, supported by DFT-based geometry optimization of the ligand, was employed to explore its potential molecular interactions with biofilm-related bacterial proteins.

## 2. Results

### 2.1. Chemical Composition of Achillea Millefolium Essential Oil and the Isolation of Borneol

*A. millefolium* EO is a transparent, mobile liquid of a bluish-green color with a characteristic intense herbaceous-camphoraceous odor. The oil has a pronounced spicy-balsamic, slightly bitter odor. It is practically insoluble in water but dissolves well in organic solvents such as ethanol, diethyl ether, and chloroform. The relative density of the EO at 20 °C is typically 0.89–0.93, and the refractive index is 1.48–1.51.

**Figure 2:**
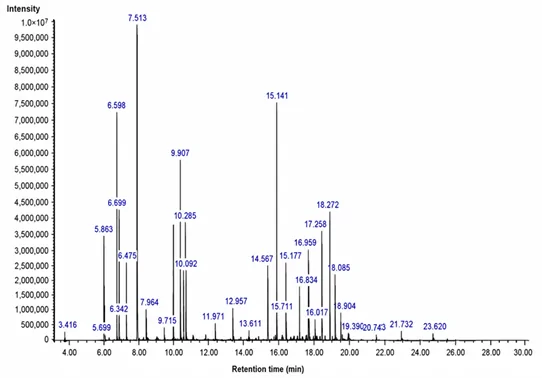
GC-MS chromatogram of *Achillea millefolium* essential oil.

The chemical composition of the EO was analyzed by gas chromatography–mass spectrometry (GC–MS), and a total of 65 components were identified, representing the major fraction of the oil (Table 1, Figure 2).

The identified compounds together accounted for almost the entire volatile fraction and mainly belonged to monoterpenes, oxygenated monoterpenes, sesquiterpenes, and oxygenated sesquiterpenes. The major constituents of the EO were eucalyptol (1,8-cineole) (14.36%), limonene oxide (9.42%), borneol (6.63%), cedrol-type alcohol (6.61%), β-pinene (8.28% total), α-bisabolol (4.72%), camphor (3.67%), and α-terpineol (3.49%). In addition, several other monoterpenes such as α-pinene (2.32%), camphene (0.67%), β-phellandrene (1.63%), and linalool (0.32%) were detected in smaller amounts.

To provide a more comprehensive characterization of the essential oil’s chemical composition, the identified compounds were grouped into the main classes of terpenes and non-terpenoid compounds (Table 1). Oxygen-containing monoterpenes accounted for the largest share (44.92%), among which eucalyptol (14.36%), limonene oxide (9.42%), borneol (6.63%), camphor (3.67%), and α-terpineol (3.49%). The second most abundant group consisted of oxygen-containing sesquiterpenes (20.14%), represented mainly by cedrol-like alcohol (6.61%), α-bisabolol (4.72%), caryophyllene oxide (2.90%), nerolidol (2.39%), and spatulene (1.51%). Monoterpene hydrocarbons accounted for 16.62%, sesquiterpene hydrocarbons for 8.96%, and non-terpenoid compounds for 9.36%.

Oxygenated monoterpenes constituted a significant proportion of the oil, particularly eucalyptol, borneol, camphor, terpinen-4-ol, and α-terpineol, which are known to contribute to the biological activities of EOs, including antimicrobial and anti-inflammatory properties. The presence of sesquiterpenes such as β-caryophyllene (2.26%), nerolidol (2.39%), spathulenol (1.51%), and caryophyllene oxide (2.90%) further indicates the complex terpene profile of the oil. Overall, the obtained results demonstrate that the EO is characterized by a monoterpene-rich composition with a predominance of oxygenated compounds, particularly 1,8-cineole and borneol derivatives, which may contribute significantly to its biological activity.

**Figure 3:**
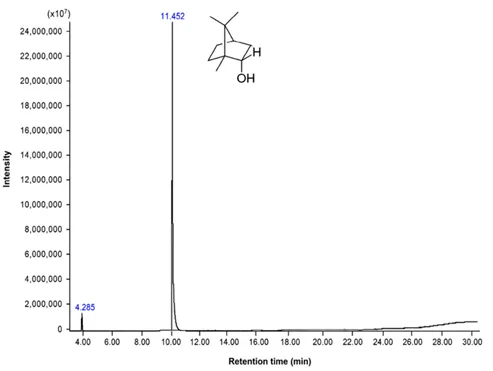
GC-MS chromatogram of isolated (+)-endo-borneol from *A. millefolium* essential oil.

*A. millefolium* EO (48 g) was subjected to vacuum fractional distillation, and the fraction with a boiling range of 89–94 °C (15 mm Hg) was collected. The resulting fraction (14.4 g) was further purified by column chromatography on SiO_2_ (sample: adsorbent ratio = 1:15) using petroleum ether as the eluent. As a result, 2.1 g of borneol was isolated. The structure of the isolated compound **10** was confirmed by GC-MS and NMR. White crystalline solid; mp 206–208 °C. [α]D^20^ +37° (c 1.0, CHCl_3_). ^1^H NMR (CDCl_3_, δ, ppm): 0.82 (s, 3H, CH_3_-7), 0.83 (s, 3H, CH_3_-9), 0.85 (s, 3H, CH_3_-10), 0.92 (dd, 1H, H-4), 1.20 (m, 2H, H-6), 1.60 (t, 1H, H-5), 1.71 (m, 1H, H-5), 1.77 (br s, 1H, OH), 1.87 (m, 1H, H-3), 2.25 (m, 1H, H-3), 3.50 (m, 1H, H-2). The spectral data were consistent with literature values for borneol. GC–MS analysis confirmed that the isolated compound was (+)-endo-borneol **10**, which accounted for approximately 98% of the chromatographic peak area, indicating high purity (Figure 3).

### 2.2. Biofilm Inhibition

**Figure 4:**
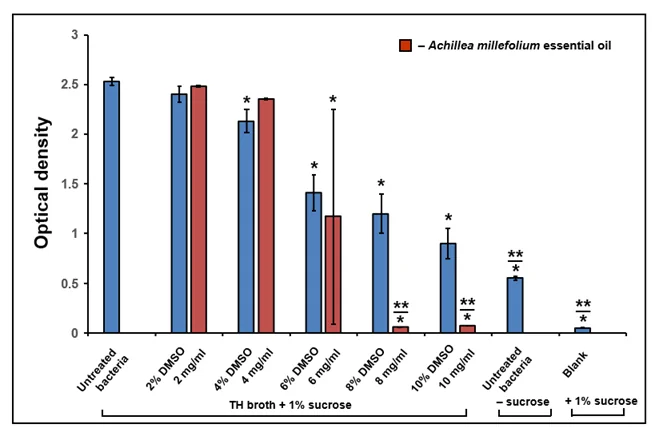
*Streptococcus mutans* biofilm density (biomass) after 24 h of incubation in the presence of *Achillea millefolium* essential oil and DMSO. * *p* < 0.05 compared to untreated bacteria +1% sucrose; ** *p* < 0.05 compared to DMSO values (*n* = 2–26) are means ±standard error from one experiment. One-Way ANOVA, LSD Post Hoc test (SPSS program, version 23.0).

The EO of *A. millefolium* exhibited inhibitory activity against biofilm formation by *S. mutans* in a concentration-dependent manner (Figure 4) The EO at concentrations of 6, 8, and 10 mg/mL reduced *S. mutans* biofilm formation by 54%, 98%, and 98%, respectively, compared with untreated bacteria (*p* < 0.05) in Todd Hewitt broth (THB) supplemented with 1% sucrose. The presence of sucrose in the medium promotes the synthesis of extracellular polysaccharides by *S. mutans*, which are essential for the formation and structural stability of dental biofilms. Since *S. mutans* plays a key role in the development of dental caries through the production of acidogenic biofilms on tooth surfaces, inhibition of its biofilm formation represents an important strategy for caries prevention. The isolated compound borneol **10** demonstrated the strongest inhibitory effect on *S. mutans* biofilm formation in the medium containing 1% sucrose.

**Figure 5:**
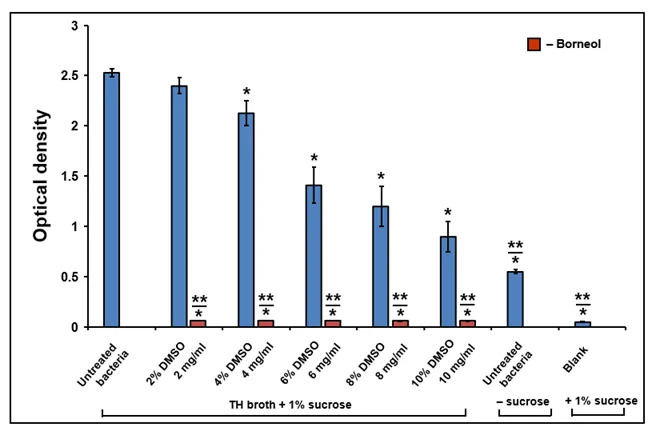
*Streptococcus mutans* biofilm density (Biomass) after 24 h of incubation in the presence of borneol and DMSO. * *p* < 0.05 compared to untreated bacteria +1% sucrose; ** *p* < 0.05 compared to DMSO values (*n* = 2–26) are means ±standard error from one experiment One-Way ANOVA, LSD Post Hoc test (SPSS program, version 23.0).

**Figure 6:**
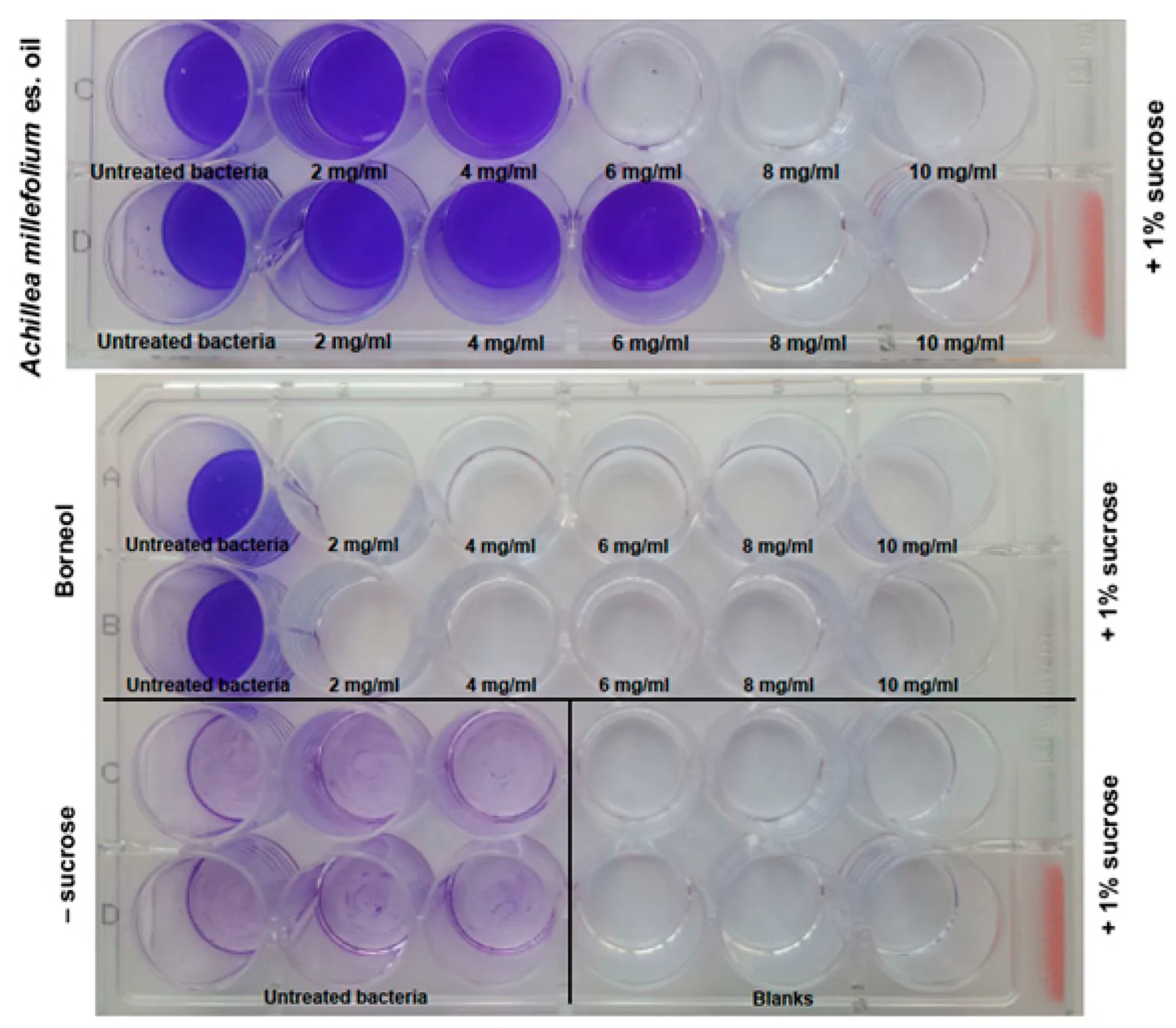
*Streptococcus mutans* biofilm stained with 0.01% crystal violet solution after 24 h of incubation in the presence of *Achillea millefolium* and borneol.

Borneol 10 at concentrations of 2, 4, 6, 8, and 10 mg/mL reduced biofilm formation by 97–98% compared with the untreated control (*p* < 0.05) (Figure 5 and Figure 6). These results suggest that borneol may be one of the key compounds responsible for the antibiofilm activity of *A. millefolium* EO. At all tested concentrations, borneol reduced *S. mutans* biofilm biomass stained with crystal violet to near-background levels (97–98%), and no concentration-dependent difference was resolved between 2 and 10 mg/mL.

The solvent dimethyl sulfoxide (DMSO) also reduced *S. mutans* biofilm formation in a concentration-dependent manner; however, the inhibitory activity of the EO and borneol was considerably greater than that observed for DMSO.

### 2.3. The Effect of Subinhibitory Concentrations of Borneol on the Expression of Virulence Genes in Streptococcus Mutans

Using the serial dilution method, the minimum inhibition concentration (MIC) and minimum bactericidal concentration (MBC) values for borneol against *S. mutans* ATCC 25175 were determined to be 2.5 mg/mL and 5 mg/mL, respectively (Table 2).

To assess the effect of borneol on the expression of virulence genes in *S. mutans*, relative gene expression was analyzed using quantitative real-time PCR (qPCR).

Expression analysis showed that exposure to subinhibitory concentrations of borneol leads to changes in the transcriptional activity of genes associated with the regulation of *S. mutans* virulence.

The yycF gene, which is part of the two-component regulatory system YycFG, demonstrated increased expression levels following exposure to borneol. At 1/4 MIC of borneol, the expression level of yycF increased by 1.62-fold compared to the control culture. Upon exposure to 1/2 MIC, an increase in expression of up to 1.48-fold was observed. The obtained data indicate the activation of regulatory systems involved in the bacterial cellular response to the antimicrobial compound.

The *gtfB* gene, which encodes glucosyltransferase B and is involved in the synthesis of extracellular glucans, also demonstrated an increase in transcription levels. Upon exposure to 1/4 MIC of borneol, gtfB expression increased approximately 1.5-fold compared to the control. At a concentration of 1/2 MIC, a moderate increase in gene expression was observed, amounting to approximately 1.3–1.4 times that of the control culture.

### 2.4. Molecular Docking Results 

For the PDB protein 6CAM, borneol exhibited negative GlideScore (−4.713 kcal/mol) and Emodel (−33.94 kcal/mol) values, indicating the formation of a thermodynamically favorable complex. However, compared to the native ligand, borneol exhibits less favorable binding parameters: the native ligand’s GlideScore was more negative (−6.371 kcal/mol), as was its Emodel (−53.861 kcal/mol) (Table 3). A similar trend was observed for LE: for borneol, LE was −0.428, whereas for the native ligand it was −0.531, indicating a lower “specific” efficiency of borneol interactions per heavy atom.

**Figure 7:**
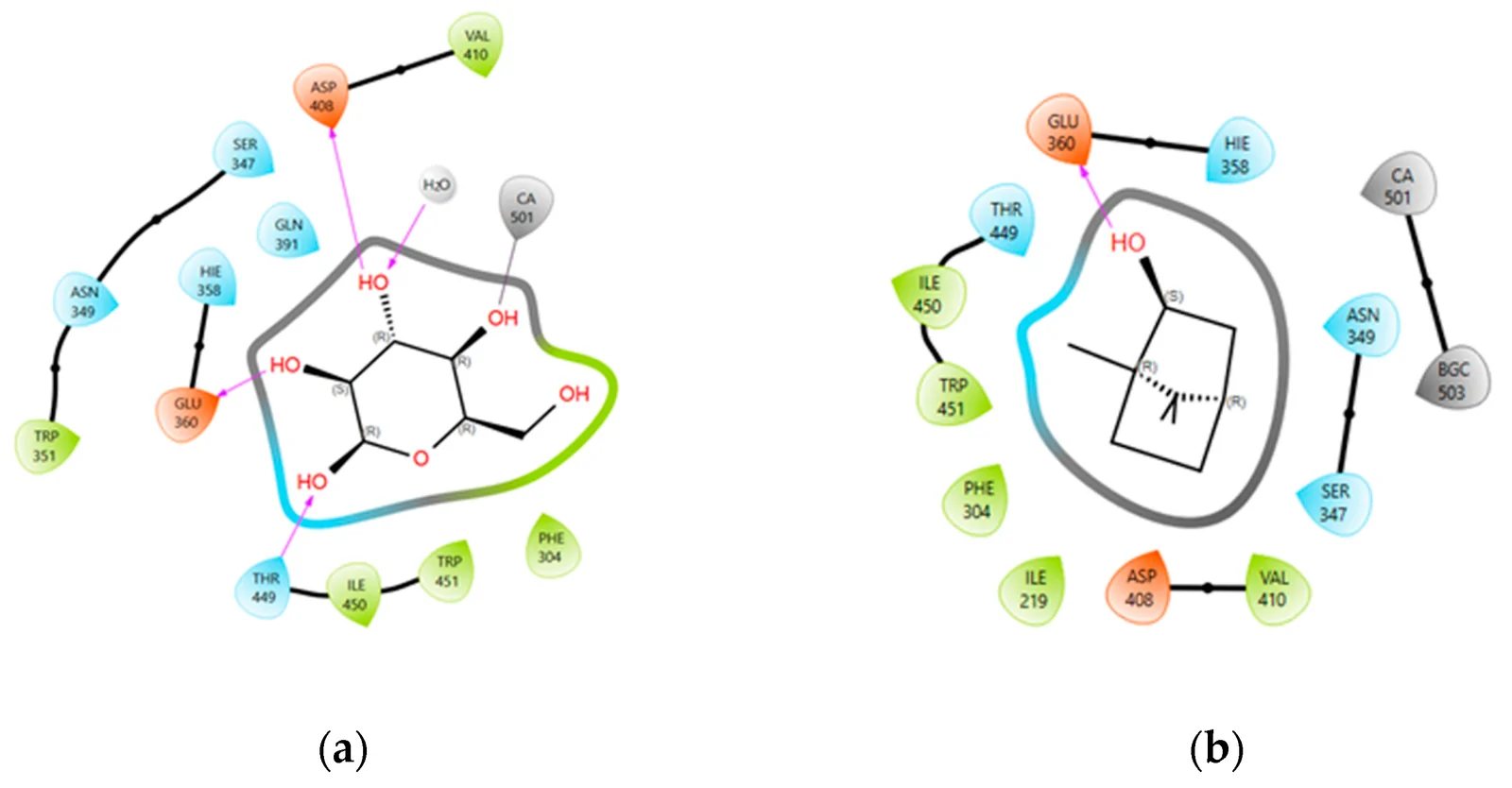
Types of noncovalent interactions: (**a**) borneol; (**b**) native ligand with protein 6 SAM. Hydrogen bonds are indicated by purple arrows.

An analysis of the interactions (Figure 7) showed that the binding of borneol to the 6CAM target is supported by a set of non-covalent contacts, including hydrogen bonds (indicated by purple arrows), likely mediated by the hydroxyl group. Overall, the obtained estimates indicate that borneol is capable of occupying the 6CAM binding site but is inferior to the native ligand in terms of predicted complex stability and optimality, which reduces the likelihood of competitive displacement of the native ligand under otherwise equal conditions.

**Figure 8:**
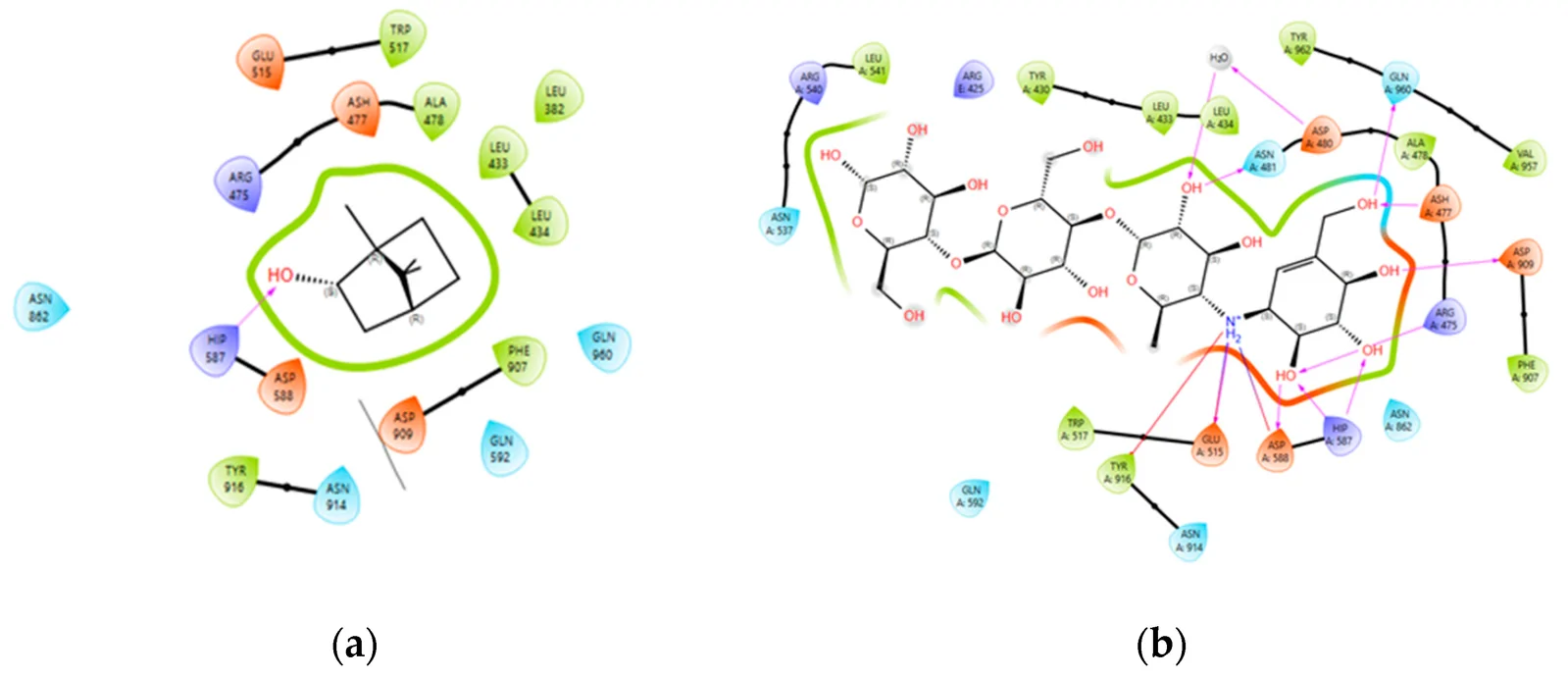
Types of noncovalent interactions between (**a**) borneol and (**b**) the native ligand with the 3AIC protein. Hydrogen bonds are indicated by purple arrows.

To further verify the reproducibility of this trend, borneol was docked against a second target (PDB: 3AIC) (Figure 8). The obtained GlideScore (−4.547 kcal/mol), LE (−0.413), and Emodel (−33.212 kcal/mol) values are close to the results for 6CAM. This indicates a binding profile of borneol that is comparable in strength and “quality” across two independent systems and is consistent with the fact that borneol complex formation is moderately favorable and is largely determined by general (non-specific) non-covalent interactions.

## 3. Discussion

EOs from medicinal plants are considered a promising source of natural inhibitors of biofilm formation by cariogenic bacteria, particularly *S. mutans*. The main biologically active components of EOs are terpenes and terpenoids, among which mono- and sesquiterpenoids exhibit the most pronounced antibacterial and anti-biofilm activity. It has been shown that phenolic monoterpenoids, such as thymol and carvacrol, are capable of inducing stress responses, cell autolysis, and significantly inhibiting the formation of *S. mutans* biofilm [23]. Their mechanism of action is associated with disruption of cell membrane integrity, inhibition of exopolysaccharide synthesis, and suppression of the activity of glucosyltransferases involved in biofilm matrix formation [33]. Farnesol, a sesquiterpene alcohol, acts as a quorum sensing signaling molecule and is capable of inhibiting glucan formation and the development of mature *S. mutans* biofilm [34]. In addition, monoterpene alcohols such as linalool, geraniol, and borneol exhibit anti-adhesive activity, reducing bacterial attachment to surfaces and decreasing the synthesis of exopolysaccharides [35]. Overall, EO terpenoids are capable of inhibiting key stages of biofilm formation, including cell adhesion, matrix synthesis, and the quorum sensing system, making them promising candidates for the development of new caries prevention agents and antimicrobial preparations for the oral cavity [36].

In our study the *gtfB* and *yycF/yycG* genes were selected for expression analysis due to their key role in regulating virulence and biofilm formation in *S. mutans* [37]. The *gtfB* gene encodes glucosyltransferase B, an enzyme that catalyzes the synthesis of insoluble glucans from sucrose. These glucans are the main component of the extracellular matrix of the biofilm and ensure the adhesion of *S. mutans* to the surface of tooth enamel, making gtfB one of the key factors in the cariogenicity of this microorganism [38]. Changes in the expression of this gene directly reflect the potential effect of the tested compounds on the ability of bacteria to form a biofilm. The two-component regulatory system YycFG (VicRK) plays an important role in regulating cell wall metabolism, cell division, and the expression of genes associated with biofilm formation in Gram-positive bacteria. The YycF protein functions as a transcriptional regulator and controls the expression of a number of virulence genes, including genes encoding extracellular polysaccharides. In particular, it has been shown that the YycFG system is involved in the regulation of glucosyltransferases, including gtfB. Thus, simultaneous analysis of the regulatory gene (*yycF*) and the virulence effector gene (*gtfB*) allows for the assessment of both changes in the bacterial signaling regulatory systems and the potential impact on biofilm formation [39,40].

It has previously been established that EO components such as camphor and 1,8-cineole have the ability to inhibit the growth of cariogenic microorganisms [41]. However, despite its prevalence in the EOs of plants of the genus *Achillea*, there is virtually no data in the literature on the anticariogenic activity of borneol. This circumstance served as the basis for isolating borneol and evaluating its activity against *S. mutans*.

Similar antibiofilm effects against *S. mutans* have been reported for other monoterpenes such as thymol, carvacrol, and eugenol, which are known to interfere with glucosyltransferase activity and biofilm matrix formation [42]. An apparently unexpected finding of the present study was the upregulation of gtfB and yycF following exposure to subinhibitory concentrations of (+)-endo-borneol. This transcriptional response should be considered separately from the pronounced reduction in biofilm biomass observed at higher borneol concentrations. Biofilm formation was evaluated at concentrations of 2–10 mg/mL using *S. mutans* UA159, whereas gene expression was assessed at 0.625 mg/mL (1/4 MIC) and 1.25 mg/mL (1/2 MIC) using *S. mutans* ATCC 25175. Therefore, these experiments represent bacterial responses under distinct exposure conditions and in different strains, precluding a direct mechanistic comparison between biofilm inhibition and transcriptional responses. Moreover, the crystal violet assay measures total attached biomass rather than bacterial viability or individual extracellular matrix components [43]. Therefore, increased gtfB and yycF transcript levels alone do not establish the mechanism underlying the observed reduction in biofilm biomass.

The increased expression of gtfB and yycF may represent a compensatory response of surviving bacterial cells to subinhibitory chemical stress. Under these conditions, bacteria may increase the transcription of genes involved in cell-envelope homeostasis, extracellular polysaccharide synthesis, and biofilm-associated adaptation. Nevertheless, this interpretation remains hypothetical. Increased mRNA abundance does not necessarily result in increased protein abundance, glucosyltransferase activity, extracellular glucan production, or biofilm biomass. Post-transcriptional regulation, protein turnover, and changes in enzymatic activity may contribute to a discrepancy between transcriptional and phenotypic responses.

Accordingly, the present results demonstrate that subinhibitory concentrations of (+)-endo-borneol modulate the transcription of gtfB and yycF, but they do not establish a causal relationship between this transcriptional response and the reduction in biofilm biomass. Further studies assessing protein expression, glucosyltransferase activity, extracellular glucan production, and biofilm architecture across matched borneol concentrations are required to clarify the biological significance of this response.

**Figure 9:**
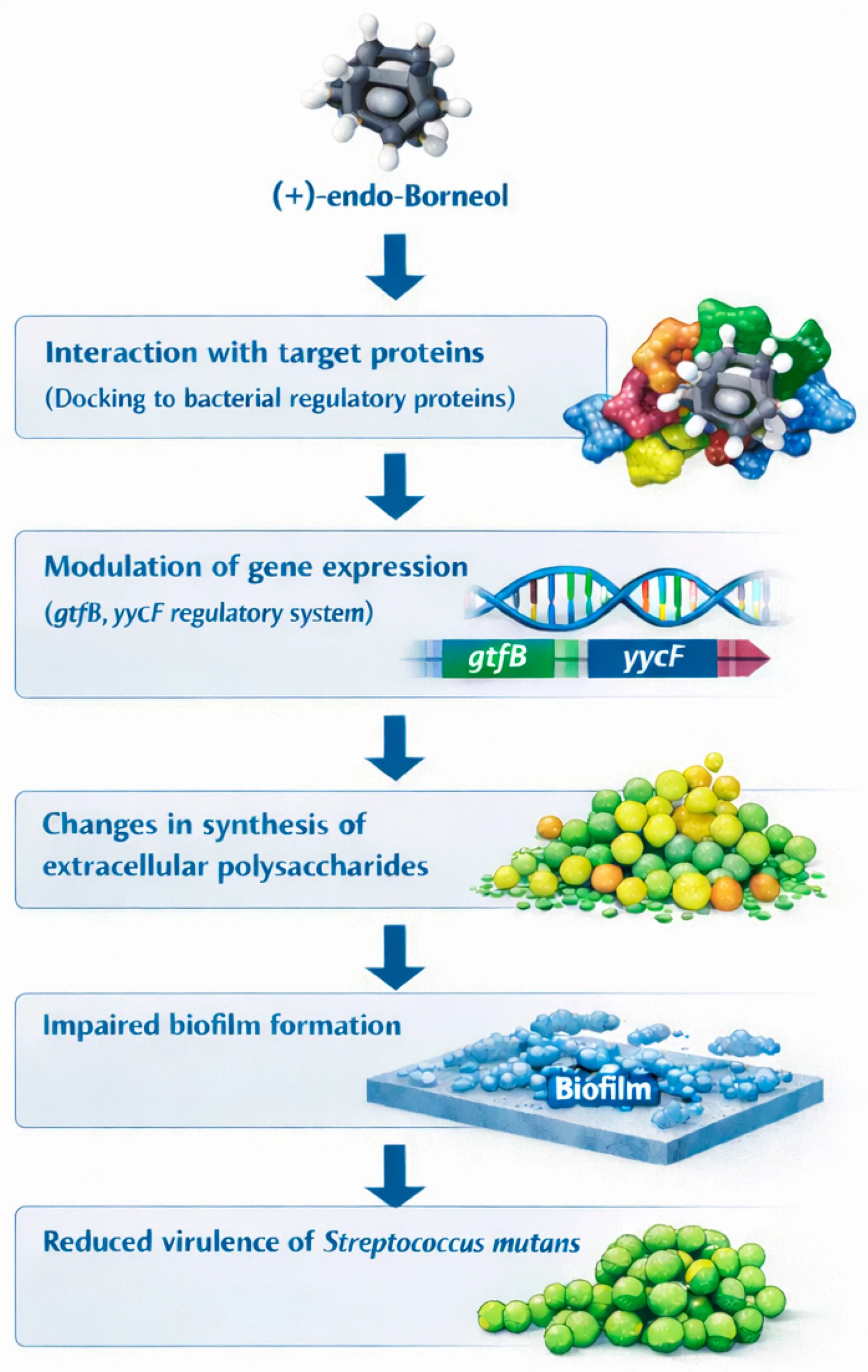
Hypothetical model of the potential antibiofilm activity of (+)-endo-borneol against *Streptococcus mutans*.

An important limitation of the present study is the absence of a standard antimicrobial or antibiofilm agent as a positive control. Therefore, although the activity of (+)-endo-borneol was evaluated relative to untreated and solvent controls, its efficacy cannot be directly compared with that of established agents such as chlorhexidine. Future studies should include appropriate positive controls to determine the relative antibiofilm potency and potential practical relevance of (+)-endo-borneol (Figure 9).

Borneol may interact with bacterial target proteins, leading to modulation of virulence-related gene expression (e.g., gtfB and yycF). These changes affect extracellular polysaccharide synthesis and biofilm formation, resulting in reduced bacterial virulence.

## 4. Materials and Methods

### 4.1. Chemicals, Bacterial Strains and Solvents

Silica gel (60–120 mesh), n-hexane and ethyl acetate were purchased from Merck (Merck Millipore, Darmstadt, Germany). Todd-Hewitt broth and agar medium (THB, Condalab, Madrid, Spain) supplemented with 1% sucrose were used for cultivation of *Streptococcus mutans*. Two *S. mutans* strains were used in this study: UA159 was employed for the biofilm inhibition assay, whereas ATCC 25175 was used for MIC/MBC determination and qPCR analysis. Cultures were incubated at 37 °C in an atmosphere containing 5% CO_2_. Strain identification was performed using MALDI-TOF mass spectrometry (Microflex-LT, Bruker Daltonics, Bremen, Germany).

**Figure 10:**
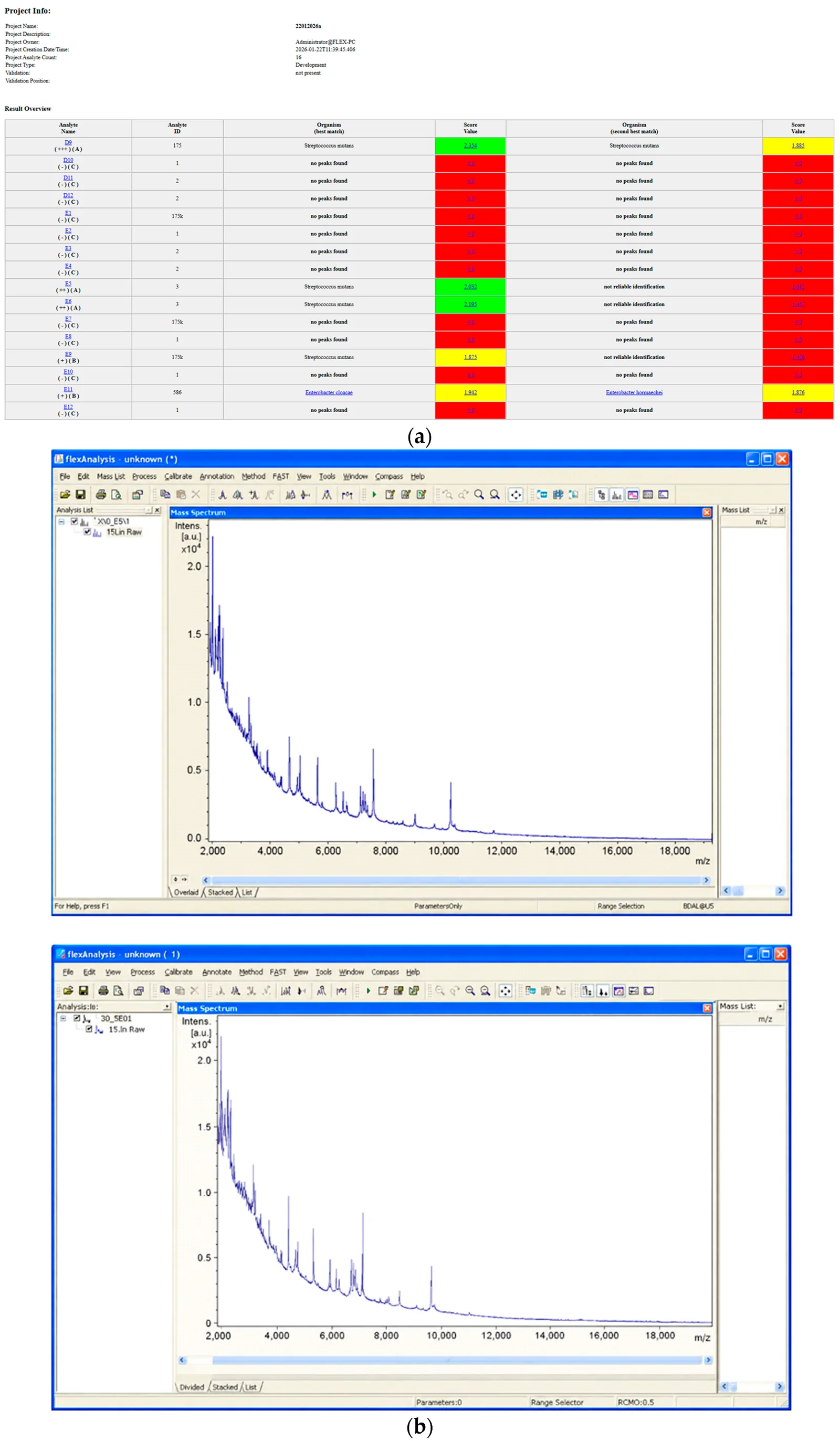
(**a**) MALDI-TOF identification report. (**b**) Representative MALDI-TOF mass spectra for measurements E5 and E6.

MALDI-TOF mass spectrometry identified the analyzed bacterial colonies as *S. mutans*, with score values of 2.032 and 2.053 for representative measurements. The corresponding identification report and mass spectra are provided in Figure 10a,b.

### 4.2. Isolation and Chemical Composition of the Essential Oil of Achillea Millefolium L. and the Isolation of Borneol

The aerial parts of wild *Achillea millefolium* L., native to the flora of Kazakhstan, were collected at the budding stage in May 2023 during field expeditions in the Karkaralinsk District, Republic of Kazakhstan (49°24′44″ N, 75°28′13″ E). The plant material was taxonomically identified by botanist M. Yu. Ishmuratova, and a voucher specimen was deposited in the Herbarium of E.A. Buketov Karaganda University under accession number QAR12576. A total of 50 g of air-dried material was subjected to hydrodistillation using a Clevenger-type apparatus for 3 h [5]. The essential oil yield was 0.85% (*w*/*w*), calculated on a dry weight basis.

GC-MS analysis of the EO and borneol was performed on an Agilent 7890A gas chromatograph (Agilent Technologies, Santa Clara, CA, USA) with an Agilent 5975C mass-selective detector under the following conditions: column type—DHA-100; column length—30 m; column diameter—0.25 mm; column packing thickness—0.25 µm; evaporator temperature—280 °C; column temperature—60–300 °C; column heating rate—6 °C/min; ion source temperature—230 °C; quadrupole condenser temperature—150 °C; carrier gas—helium; column gas pressure—2 psi; sample volume—1.0 µL; injection mode—20:1 split; mass spectrum acquisition mode—scan; library—NIST 08; method for calculating fraction composition—relative peak area (semi-quantitative method). Analysis time—42 min. Data processing was performed automatically using the GS MSD Data Analysis software, version 2.72.

Borneol was isolated from *A. millefolium* EO obtained from the aerial parts of the plant by hydrodistillation in a Clevenger apparatus. To isolate the individual compound, the EO was fractionated by column chromatography on silica gel (60–120 mesh). Chromatographic separation was performed using an n-hexane–ethyl acetate solvent gradient system with a gradual increase in eluent polarity. The obtained fractions were monitored by thin-layer chromatography on silica gel plates (Silica gel 60 F254) with visualization of spots under UV light and after treatment with developing reagents. Fractions with identical chromatographic profiles were combined and subjected to further purification.

The compound corresponding to borneol was isolated as a single component after repeated chromatographic purification. The compound under study was identified using gas chromatography with mass spectrometry detection (GC-MS) by comparing the experimental mass spectrum with the NIST08 library. The main peak on the chromatogram with a retention time of 11.452 min was identified as endo-borneol.

The compound with a retention time of 11.453 min is characterized by a major ion of *m*/*z* 95.1, as well as ions of *m*/*z* 41.1 and 139.1. Based on a search of the NIST08 library, the best match was obtained for endo-borneol (Quality = 97), and the second-best match was for endo- (Quality = 94), which confirms the identification of the compound under investigation.

### 4.3. Inhibition of Streptococcus mutans Biofilm Formation by Achillea millefolium Essential Oil and Borneol Assay

Inhibition of *S. mutans* biofilm formation by *A. millefolium* EO and borneol was conducted according to the method described in [21]. *S. mutans* (strain UA159) stock in skim milk was thawed, and 10 μL of its suspension was inoculated to the starter culture vials containing 990 µL of THB. Then, the starter culture vials were incubated anaerobically (95% N_2_ + 5% CO_2_) at 37 °C for 18 h. For purity check, the loopful of *S. mutans* stock suspension was inoculated to Columbia agar plates with 7% sheep blood. Then, the plates were incubated anaerobically (95% N_2_ + 5% CO_2_) at 37° C for 48 h. After 18 h, optical density (OD) adjustment of starter cultures was performed in 1:5 dilutions in 96-well microplate using the Dynex MRX^™^ microplate-reader spectrophotometer (Synergy HTX, BioTek Instrument Inc., Winooski, VT, USA) at 630 nm. THB without and with 1% sucrose was dispensed into 24-well, flat-bottomed, polystyrene tissue culture plates (Falcon, Corning Incorporated, Corning, NY, USA) (the final volume of liquid per well was 1 mL). The stock concentrations of EOs (100 mg/mL) and borneol (100 mg/mL) were prepared in the pure DMSO. The EO *A. millefolium* and borneol were added to the plates at the final concentrations of 2, 4, 6, 8 and 10 mg/mL, and DMSO was added to the plates at the final concentrations of 2, 4, 6, 8 and 10%.

The plate wells were inoculated with *S. mutans* starter culture at the final dilution of 1:100, and the plates were incubated anaerobically (95% N2 + 5% CO_2_) at 37 °C for 24 h. The plate wells were rinsed with distilled water in order to remove loosely bound bacterial cells, and then the biofilm in bottom of wells was fixed with 95% ethanol. The biofilm was stained with 0.01% crystal violet solution, and then the bound dye was extracted using 33% acetic acid solution. Afterwards, the OD of samples was measured using Dynex MRX™ microplate-reader spectrophotometer at 595 nm.

### 4.4. Determination of the MIC and MBC of Borneol

MIC and MBC of borneol against *S. mutans* ATCC 25175 cells were determined in accordance with the recommendations of the Clinical and Laboratory Standards Institute (CLSI) and ISO 20776-1: 2019 using the serial dilution microplate method in broth [44,45].

Borneol was dissolved in Dimethyl sulfoxide (DMSO; HPLC grade; Fisher Chemical; product code D/4125/PB07) and tested in a range of final concentrations from 10 mg/mL to 39 μg/mL. A 100 mg/mL borneol stock solution was prepared in pure DMSO. The final bacterial inoculum was adjusted to 5 × 10^5^ CFU/mL, and the final volume in each well was 150 μL. The final DMSO concentrations corresponding to the MIC and MBC of (+)-endo-borneol were 0.42% (*v*/*v*) and 0.83% (*v*/*v*), respectively. Concentration-matched DMSO controls were included to verify that the solvent itself did not affect bacterial growth. A bacterial suspension was added to the plate wells and incubated at 37 °C in a 5% CO_2_ atmosphere for 24 h.

To verify the accuracy of the method, the following control samples were used: a negative control (culture medium with borneol but no bacterial culture), a positive control (culture medium with a bacterial culture), and a solvent control (culture medium with a bacterial culture and DMSO). During preliminary experiments, it was established that DMSO at the initial concentration exhibits its own antibacterial activity, as evidenced by the absence of microbial growth in the solvent control well. Therefore, to exclude the influence of the solvent on the experimental results, a series of sequential dilutions of DMSO was prepared to determine the concentration that did not exert an inhibitory effect on the test microorganism. In subsequent experiments, a concentration of DMSO that did not affect bacterial growth was used.

The MIC was defined as the lowest concentration of the drug that inhibited visible bacterial growth. The MBC was defined as the minimum concentration at which no colony growth was observed after plating on agar plates.

### 4.5. Total RNA Extraction, Reverse Transcription, and Quantitative Real-Time PCR Analysis

To assess the effect of borneol on the expression of the *gtfB* and *YycF* genes, a 24 h culture of *S. mutans* ATCC 25175 was incubated for 24 h at various concentrations of borneol (1/2 MIC–1.25 mg/mL and 1/4 MIC–0.625 mg/mL). After incubation, the cells were washed with phosphate-buffered saline to remove residual borneol.

Total RNA extraction and subsequent storage were performed according to the manufacturer’s instructions using the Aurum Total RNA Mini Kit (Bio-Rad Laboratories, Hercules, CA, USA). RNA concentration and integrity (RIN) were assessed using a NanoPhotometer P330 spectrophotometer (IMPLEN, GmbH, Munich, Germany).

The High-Capacity cDNA Reverse Transcription Kit (Applied Biosystems, USA) was used for cDNA synthesis. Custom TaqMan Gene Expression Assays were manufactured by Applied Biosystems (Thermo Fisher Scientific, Waltham, MA, USA)and supplied as pre-formulated 20× mixtures containing gene-specific forward and reverse primers and a fluorescently labelled TaqMan probe with a nonfluorescent quencher. The primer sequences used for the qPCR analysis are presented in Table 4. The gtfB and yycF assays contained FAM-labelled probes, whereas the reference gyrB assay was supplied as a VIC-labelled, primer-limited assay. Quantitative PCR was performed using the Custom TaqMan Gene Expression Assay (Thermo Fisher Scientific, Waltham, MA, USA) on the Applied Biosystems QuantStudio 5 Real-Time PCR System (Thermo Fisher Scientific, Waltham, MA, USA). A 10 μL reaction mixture contained 5 μL of TaqMan Gene Expression Master Mix (2×), 1 μL of cDNA, 0.5 μL of TaqMan assay, and 3.5 μL of nuclease-free water. In accordance with the manufacturer’s instructions, the qPCR protocol included the following steps: uracil-N-glycosylase incubation (2 min at 50 °C, 1 cycle), polymerase activation (10 min at 95 °C, 1 cycle), denaturation (40 cycles of 15 s at 95 °C), and annealing/extension (40 cycles of 1 min at 60 °C). Fluorescence was detected at the end of each cycle. The expression levels of the *gtfB* and *YycF* genes were normalized relative to the GyrB gene, which was used as an endogenous control. A bacterial culture grown without the addition of borneol was used as a control group. After incubation, total RNA was isolated from the bacterial cells, followed by cDNA synthesis. Quantitative assessment of gene expression was performed by qPCR using specific primers for the *gtfB* and *yycF* genes. The *gyrB* gene, encoding a DNA gyrase subunit, was used as a reference gene, which allowed for the normalization of the transcription levels of the genes under study.

Relative expression levels were calculated using the 2^−ΔΔCt method, where:

− ΔCt was defined as the difference between the Ct values of the target gene and the reference gene (*gyrB*);
− ΔΔCt was calculated relative to the control group.

The control expression value was set to 1, and changes in expression in the experimental groups were expressed as a fold change (RQ).

Quantitative analysis was performed using Design and Analysis 2 software, version 2.6.0 (Thermo Fisher Scientific, USA), followed by data analysis using the ΔΔCt method.

### 4.6. Molecular Docking of Borneol

Molecular modeling was performed using the Schrödinger Small Molecule Drug Discovery Suite 2024-3 [46] software package (Schrödinger, LLC, New York, NY, USA), applying the Induced Fit Docking (IFD; Glide [47] and Prime [48,49]), which ensures that the amino acid residues of the binding site adapt to the ligand under study. Two proteins from *Streptococcus mutans*, which play a key role in biofilm formation, were selected as protein targets: the glucan-binding protein (GbpC, PDB ID:6CAM), involved in bacterial adhesion and biofilm structure stabilization, and the glucosyltransferase (GtfC, PDB ID: 3AIC), which catalyzes the synthesis of extracellular glucans that form the polysaccharide matrix of the biofilm. The crystal structures of the proteins were obtained from the Protein Data Bank and prepared for docking using the standard Protein Preparation Wizard (Schrödinger Release 2024-3, Schrödinger, LLC, New York, NY, USA), which includes the addition of hydrogen atoms, the assignment of chemical bond orders, the optimization of protonation states of amino acid residues at physiological pH, and a limited energy minimization. The geometry of the compound under study was optimized using the DFT B3LYP/6-31G** [50] quantum-chemical method using the Gaussian 16W program Rev. C.01 (Gaussian, Inc., Wallingford, CT, USA) [51]. Docking was performed in Glide XP (Glide, Schrödinger Release 2024-3, Schrödinger, LLC, New York, NY, USA) mode using flexible models of the protein and ligand; the docking grid was generated relative to the binding site of the co-crystallized ligand, and the docking region size was 15 Å. GlideScore, Ligand Efficiency (LE), and the Emodel integral energy parameter were used to rank the obtained conformations. As an internal control, the docking results for (+)-endo-borneol were compared with the binding parameters of the co-crystallized (native) ligand for each protein target.

### 4.7. Statistical Analysis

The biofilm assay was performed as one independent experiment (biological *n* = 1), with multiple technical replicate wells for each condition. Results are presented as the mean ± standard error of the mean (SEM) of technical replicates. Homogeneity of variance was assessed using Levene’s test, whereas normality was not formally evaluated. Data were analysed using one-way ANOVA (IBM SPSS Statistics for Windows, version 23.0 (IBM Corp., Armonk, NY, USA)). followed by the least significant difference (LSD) post hoc test. A *p*-value < 0.05 was considered statistically significant. Analyses were performed using IBM SPSS Statistics for Windows, version 23.0 (IBM Corp., Armonk, NY, USA).

## 5. Conclusions

Subinhibitory concentrations of (+)-endo-borneol did not suppress the expression of the biofilm-associated genes *gtfB* and *yycF*. Instead, moderate transcriptional upregulation was observed. This response may reflect compensatory adaptation to subinhibitory chemical stress; however, its biological significance remains uncertain because protein expression, glucosyltransferase activity, and extracellular glucan production were not evaluated.

Molecular docking predicted potential interactions between (+)-endo-borneol and selected proteins associated with bacterial adhesion and extracellular glucan synthesis. However, these computational findings do not confirm direct protein binding, inhibition of enzymatic activity, or a causal mechanism of biofilm reduction. Further biochemical and functional studies are required to validate these predicted interactions and determine their biological relevance.

Of particular importance is the natural origin of the compound under study. The flora of Kazakhstan is characterized by high biodiversity and is a valuable source of medicinal plants rich in EOs and terpenoid compounds. Species of the genus *Achillea*, widely distributed throughout Kazakhstan, contain a complex of biologically active monoterpenes, including camphor, 1,8-cineole, and borneol, which possess antimicrobial properties. The study of individual components of the EOs of these plants allows for the identification of new natural compounds with a targeted effect on cariogenic microorganisms and expands the possibilities for the development of phytogenic antimicrobial agents.

Taken together, the present findings demonstrate that (+)-endo-borneol reduces *S. mutans* biofilm biomass at higher concentrations and modulates the transcription of selected biofilm-associated genes under subinhibitory exposure. While these findings support its antibiofilm potential, they do not establish a direct molecular mechanism or causal relationship between transcriptional changes and biofilm inhibition. Further biochemical, protein-level, and functional studies are required to validate the predicted targets and clarify the mechanism of action.

## Figures and Tables

**Table 1:** Chemical composition of the *Achillea millefolium* essential oil identified by GC–MS.

No	RT (min)	Compound	CAS No.	Area (%)
1	3.318	1,4-Cyclohexadiene, 1-methyl-	4313-57-9	0.19
2	4.165	(E)-2-Hexenal	6728-26-3	0.13
3	5.694	Bicyclo [3.1.0]hex-2-ene, 2-methyl-5-(1-methylethyl)	2867-05-2	0.13
4	5.859	α-Pinene	80-56-8	2.32
5	6.112	Camphene	79-92-5	0.67
6	6.343	1-Octen-3-ol	3391-86-4	0.63
7	6.475	β-Phellandrene	555-10-2	1.63
8	6.596	β-Pinene	127-91-3	5.39
9	6.696	β-Pinene	127-91-3	2.89
10	7.235	(+)-4-Carene	29050-33-7	0.41
11	7.290	p-Cymene	99-87-6	0.94
12	7.510	Eucalyptol (1,8-cineole)	470-82-6	14.36
13	7.697	3,7-Dimethyl-1,3,6-octatriene	502-99-8	0.25
14	7.961	1-Methyl-4-(1-methylethyl)-1,4-cyclohexadiene	99-85-4	1.04
15	8.060	cis-β-Terpineol	7299-40-3	0.12
16	8.478	trans-2,7-Dimethyl-3,6-octadien-2-ol	38092-33-0	0.19
17	8.522	(+)-4-Carene	29050-33-7	0.21
18	8.566	Linalool	78-70-6	0.32
19	9.017	4-Acetyl-1-methylcyclohexene	6090-09-1	0.16
20	9.061	2-Cyclohexen-1-ol, 1-methyl-4-(1-methylethyl)	29803-81-4	0.16
21	9.171	1,7-Octadien-3-one, 2-methyl-6-methylene	41702-60-7	0.23
22	9.380	Camphor	76-22-2	3.67
23	9.545	1,5,7-Octatrien-3-ol, 2,6-dimethyl	29414-56-0	0.52
24	9.776	2,6-Octadienal, 3,7-dimethyl- (citral isomer)	106-26-3	0.69
25	9.908	Borneol	464-45-9	6.63
26	10.095	Terpinen-4-ol	562-74-3	1.78
27	10.282	α-Terpineol	98-55-5	3.49
28	10.392	Myrtenol	19894-97-4	0.30
29	10.557	2-Cyclohexen-1-ol derivative	1197-06-4	0.38
30	11.250	Carvone derivative	89-81-6	0.67
31	11.976	Bornyl acetate	76-49-3	0.51
32	12.956	Eugenol	97-53-0	0.77
33	13.825	α-Cubebene	17699-14-8	0.15
34	13.979	Sesquiterpene hydrocarbon	5208-59-3	0.27
35	14.562	β-Caryophyllene	87-44-5	2.26
36	15.145	Limonene oxide (trans)	6909-30-4	9.42
37	15.277	1,5,7-Octatrien-3-ol, 3,7-dimethyl)-4-methyl-	644-30-4	0.56
38	15.475	Epi-bicyclosesquiphellandrene	54324-03-7	1.21
39	15.662	Benzene derivative	3944-08-9	1.32
40	15.739	Sesquiterpene hydrocarbon	495-61-4	0.26
41	16.025	1,5,7-Octatrien-3-ol, 3,7-dimethyl	29957-43-5	0.92
42	16.157	Sesquiterpene hydrocarbon	3242-08-8	0.29
43	16.311	Dodecanoic acid	143-07-7	0.25
44	16.454	Nerolidol	142-50-7	2.39
45	16.707	Bicyclo [7.2.0]undec-4-ene, 4,11,11-trimethyl-8-methylene-	13877-93-5	0.32
46	16.839	Spathulenol	77171-55-2	1.51
47	16.960	Caryophyllene oxide	1139-30-6	2.90
48	17.048	Sesquiterpene ketone	90054-46-9	0.26
49	17.136	Bisabolene epoxide	1000131-71-2	0.29
50	17.257	Naphthyridine derivative	60058-16-4	2.88
51	17.411	Phenol derivative	3096-71-7	0.63
52	17.499	Bisabolene epoxide	1000131-71-1	0.44
53	17.576	Himachalene derivative	4630-07-3	0.56
54	17.620	Azulene derivative	489-40-7	0.35
55	17.664	10,10-Dimethyl-2,6-dimethylenebicyclo [7.2.0]undecan-5.beta.-ol	19431-80-2	0.79
56	17.741	Not detected	6750-60-3	1.42
57	17.907	Cedrol-type alcohol	473-15-4	6.61
58	18.083	Vatirenene derivative	1000293-04-2	2.63
59	18.270	α-Bisabolol	515-69-5	4.72
60	18.325	Monoterpene hydrocarbon	5113-87-1	0.74
61	18.908	Azulene, 7-ethyl-1,4-dimethyl-	529-05-5	0.66
62	19.392	Aromadendrene oxide	17429-27-5	0.23
63	20.745	Cyclohexadecane	295-65-8	0.13
64	21.735	n-Hexadecanoic acid	57-10-3	0.57
65	23.617	Phytol	150-86-7	0.28

**Table 2:** Minimum inhibition concentration and minimum bactericidal concentration values for borneol against *S. mutans* ATCC 25175.

Name	MIC, mg/mL	MBC, mg/mL
Borneol	2.5	5

**Table 3:** Scoring parameters of molecular docking.

Ligand	Protein PDB ID	Docking Parameters, kcal/mol		
		GlideScore	LE	Emodel
Borneol	6CAM	−4.713	−0.428	−33.94
Native ligand	6CAM	−6.371	−0.531	−53.861
Borneol	3AIC	−4.547	−0.413	−33.212

**Table 4:** Primer sequences used for the qPCR analysis.

Target Gene	Forward Primer Sequence (5′–3′)	Reverse Primer Sequence (5′–3′)
*gtfB*	AGCAATGCAGCCAATCTACAAT	ACCAACTTTGCCGTTATTGTCA
*yycF*	CGAAAGAAGACATCATCGGATATTAC	CGATTAGACCTTCTTCTTCATTTAAATCT
*gyrB*	GCACAAGAGTACGATGCCAGT	TCCCAAACAAGGTGATGCAGC

## Data Availability

All the data is available from the authors and shall be provided upon request.

## Abbreviations

ANOVA	Analysis of Variance
ATCC	American Type Culture Collection
CLSI	Clinical and Laboratory Standards Institute
DFT	Density Functional Theory
DMSO	Dimethyl Sulfoxide
EO	Essential Oil
GC	Gas Chromatography
MS	Mass Spectrometry
LE	Ligand Efficiency
MALDI	Matrix-Assisted Laser Desorption/Ionization
TOF MS	Time-of-Flight Mass Spectrometry
MBC	Minimum Bactericidal Concentration
MIC	Minimum Inhibitory Concentration
NMR	Nuclear Magnetic Resonance
OD	Optical Density
qPCR	Quantitative Real-Time Polymerase Chain Reaction
RIN	RNA Integrity Number
RT	Retention Time
SD	Standard Deviation
SPSS	Statistical Package for the Social Sciences
THB	Todd–Hewitt Broth
UV	Ultraviolet
